# RNA modifications in the progression of liver diseases: from fatty liver to cancer

**DOI:** 10.1007/s11427-023-2494-x

**Published:** 2024-05-27

**Authors:** Simiao Li, Wajahat Z Mehal, Xinshou Ouyang

**Affiliations:** Department of Internal Medicine, Section of Digestive Diseases, Yale University School of Medicine, New Haven CT 06520, USA

**Keywords:** m^6^A RNA modification, NASH, NAFLD, HCC, fatty liver

## Abstract

Non-alcoholic fatty liver disease (NAFLD) has emerged as a prominent global health concern associated with high risk of metabolic syndrome, and has impacted a substantial segment of the population. The disease spectrum ranges from simple fatty liver to non-alcoholic steatohepatitis (NASH), which can progress to cirrhosis and hepatocellular carcinoma (HCC) and is increasingly becoming a prevalent indication for liver transplantation. The existing therapeutic options for NAFLD, NASH, and HCC are limited, underscoring the urgent need for innovative treatment strategies. Insights into gene expression, particularly RNA modifications such as *N*^6^ methyladenosine (m^6^A), hold promising avenues for interventions. These modifications play integral roles in RNA metabolism and cellular functions, encompassing the entire NAFLD-NASH-HCC progression. This review will encompass recent insights on diverse RNA modifications, including m^6^A, pseudouridine (Ψ), *N*^1^-methyladenosine (m^1^A), and 5-methylcytidine (m^5^C) across various RNA species. It will uncover their significance in crucial aspects such as steatosis, inflammation, fibrosis, and tumorigenesis. Furthermore, prospective research directions and therapeutic implications will be explored, advancing our comprehensive understanding of the intricate interconnected nature of these pathological conditions.

## Introduction

Non-alcoholic fatty liver disease (NAFLD) has emerged as a substantial global public health concern, impacting approximately 25% of the worldwide population, and has increasingly become a public health concern in recent years alongside obesity and metabolic syndrome (Huang et al., 2021). NAFLD comprises a spectrum of liver conditions characterized by the excessive accumulation of fat within the hepatic tissue, spanning from simple steatosis to the more severe non-alcoholic steatohepatitis (NASH). Marked by inflammation and fibrosis, NASH can potentially culminate in cirrhosis and hepatocellular carcinoma (HCC), and has become an increasingly prevalent indication for liver transplantation (Ipsen et al., 2018). Notably, NAFLD has taken the lead as the fastest-growing cause of HCC (Huang et al., 2021), with a model projecting a staggering 130% increase in NAFLD-related HCC cases in the US, from 10,820 cases in 2016 to an estimated 24,860 cases by 2030 (Estes et al., 2018).

Dietary and lifestyle modifications can ameliorate NAFLD, but there remains a dearth of pharmaceutical treatments for both NAFLD and NASH (Powell et al., 2021). First-line therapies for HCC include Sorafenib and Lenvatinib, but provide a response in a minority of patients (Yang et al., 2023a). Ultimately, understanding the molecular mechanisms underlying the progression from NAFLD to HCC is pivotal to advancing treatment options for these interconnected disorders.

A comprehensive grasp of gene expression at both transcriptional and translational levels is essential for advancing treatment strategies. Epigenetics encompasses histone modifications, DNA methylation, and chromatin rearrangement. Meanwhile, the burgeoning field of RNA modifications, known as “epitranscriptomics,” scrutinizes post-transcriptional gene regulation. RNA modification is observed across various RNA species, including mRNA, lncRNA, miRNA, circRNA, tRNA, and rRNA. Diverse modifications such as *N*^6^ methyladenosine (m^6^A), pseudouridine (Ψ), *N*^1^-methyladenosine (m^1^A), 5-methylcytidine (m^5^C), *N*^6^,2′-*O*-dimethyladenosine (m^6^Am), inosine, *N*^4^-acetylcytidine (ac^4^C), 2′-*O*-methylated nucleotide (Nm), and internal *N*^7^-methylguanosine (m^7^G) play multifaceted roles in RNA metabolism, influencing splicing, translation, stability, and export. Given the pivotal regulatory roles of RNA modifications in cellular activity, they hold potential as exciting targets for various diseases, including NAFLD, NASH, and HCC (Roundtree et al., 2017a).

In this review, we delve into the intricate roles of RNA modifications throughout the NAFLD-NASH-HCC progression. The exploration begins with an overview of the fundamental mechanisms of m^6^A methylations, subsequently revealing their impact on crucial aspects including steatosis, inflammation, fibrosis, and tumorigenesis. Finally, we contemplate the broader spectrum of RNA modifications and anticipate the future research pathways that will further illuminate this dynamic field.

### m^6^A RNA modification

m^6^A is one of the most common and well-studied RNA modifications in eukaryotes. It is a dynamic and reversible modification typically installed on the motif RRACH (R=A or G; H=A, C, or U). The m^6^A modifications can be installed by methyltransferases (writers), removed by demethylases (erasers) and recognized by RNA-binding proteins (readers).

### Writers, erasers, and readers

m^6^A modifications are primarily installed by the methyltransferase complex, which is localized in nuclear specks. The core of this complex is composed of a heterodimer between methyltransferase-like3 (METTL3) and methyltransferase-like14 (METTL14), with METTL3 being the catalytic subunit (Liu et al., 2014; Wang et al., 2016). Wilm’s tumor 1-associating protein (WTAP) is a regulatory subunit of the methyltransferase complex (Ping et al., 2014). Other m^6^A writers form the regulatory component of the methyltransferase as well, including vir like m^6^A methyltransferase associated (VIRMA), zinc finger CCCH domain-containing protein 13 (ZC3H13), E3 ubiquitin-protein ligase Hakai (HA-KAI), and RNA binding motif protein 15/15B (RBM15/15B) (Patil et al., 2016; Růžička et al., 2017; Wen et al., 2018; Yue et al., 2018). Additionally, METTL16 can install m^6^A modifications to specific motifs independently of the classic methyltransferase complex (Mendel et al., 2021).

Fat mass and obesity-associated protein (FTO) and alkB homolog 5 (ALKBH5) are the two m^6^A erasers characterized so far, and they can remove m^6^A markers from mRNAs independently of each other (Jia et al., 2011; Zheng et al., 2013).

**Figure 1.**
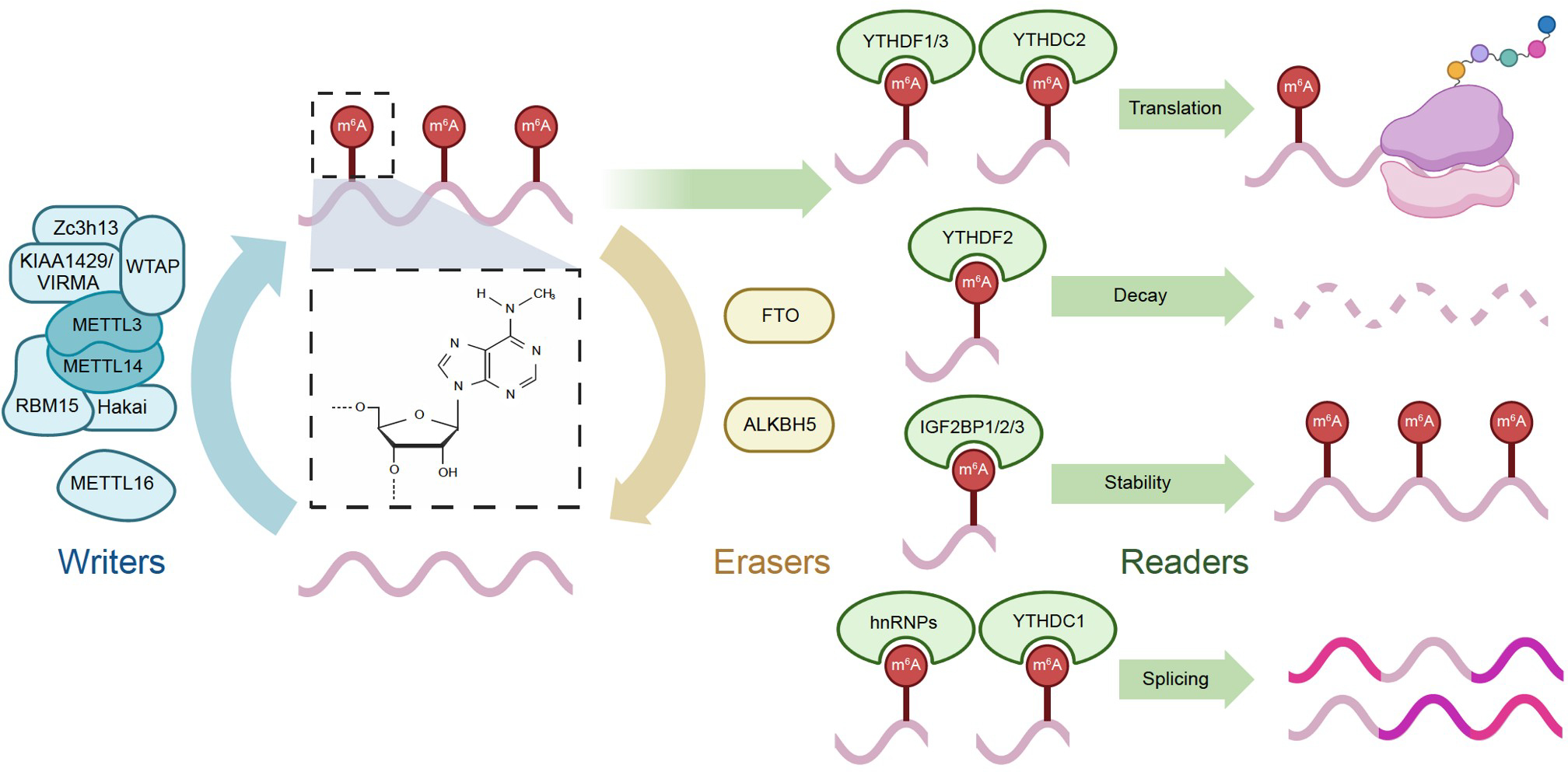
Fundamental mechanisms of m^6^A modifications. The writers such as METTL3, METTL14, and WTAP add m^6^A modifications to RNA, and the erasers, FTO and ALKBH5, remove it. Various readers, YTHDF1/2/3, YTHDC1/2, IGFBP1/2/3, and hnRNPs, recognize RNA with m^6^A modifications and promote its translation, decay, stability, or splicing.

A multitude of m^6^A readers are engaged in diverse roles in mediating RNA stability, decay, and translation. These readers can be categorized into three main groups: proteins featuring the YT521-B homology (YTH) domain, insulin-like growth factor 2 binding proteins (IGF2BPs), and heterogeneous nuclear ribonucleoproteins (hnRNPs). YTHDF2 promotes the degradation of its target transcripts, whereas YTH *N*^6^-methyladenosine RNA binding protein F1 (YTHDF1) and YTH *N*^6^-methyladenosine RNA binding protein F3 (YTHDF3) promote translation (Du et al., 2016; Shi et al., 2017). YTH *N*^6^-methyladenosine RNA binding protein C1 (YTHDC1) mediates different RNA fates including nuclear export, alternative splicing, RNA stabilization, and RNA decay, and YTH *N*^6^-methyladenosine RNA binding protein C2 (YTHDC2) regulates translation and stability (Roundtree et al., 2017b; Zhou et al., 2021). insulin-like growth factor 2 binding protein 1/2/3 (IGF2BP1, 2, and 3) all appear to stabilize their target transcripts (Bell et al., 2013; Huang et al., 2018). Finally, heterogeneous nuclear ribonucleoprotein C (HNRNPC), heterogeneous nuclear ribonucleoprotein G (HNRNPG), and heterogeneous nuclear ribonucleoprotein A2/B1 (HNRNPA2B1) regulate RNA splicing and processing (Alarcón et al., 2015). (Figure 1)

m^6^A modifications have been shown to be implicated in diverse developmental and pathological processes. It is essential to hemopoiesis, immunity, the central nervous system, the immune system, the reproductive system, and adipose tissue development and homeostasis (Ben-Haim et al., 2015; Lence et al., 2016; Vu et al., 2017; Wu et al., 2023; Zhang et al., 2017). The dysregulation of m^6^A, notably the upregulation of METTL3, has been associated with a range of cancers (Liu et al., 2023b; Vu et al., 2017; Xiang et al., 2017; Yang et al., 2019). Additionally, m^6^A modifications are implicated in metabolic diseases including diabetes mellitus, cardiovascular diseases, obesity, and notably NAFLD (De Jesus et al., 2019; Jiang et al., 2021a; Li et al., 2023; Yang et al., 2020). Taken together, in view of the increased focus on exploring the intricate interplay between m^6^A and liver diseases, a comprehensive review dedicated to this subject is not only timely but also imperative for the advancing of this field.

### The function of m^6^A in fatty liver progression

Dysregulation of m^6^A writers, readers, and erasers drives the progression of NAFLD through a variety of pathways. Cheng et al. (2022) observed elevated expression levels of METTL3, METTL14, FTO, and eukaryotic translation initiation factor 3 subunit H (EIF3H), along with decreased expression levels of WTAP, RBM15, YTHDC1, YTHDC2, IGF2BP1, HNRNPC, and HNRNPA2B1 in liver biopsy tissues from NAFLD patients. Another study found significant changes in m^6^A modification levels of 226 differentially expressed genes in high fructose diet induced NAFLD mice. Of these 226 genes, 193 were more methylated, suggesting that greater m^6^A modifications correlate with NAFLD (Luo et al., 2021). However, the amount and effect of m^6^A modifications in the NAFLD-NASH-HCC progression change based on cell types and mRNA targets.

#### Lipid metabolism regulation by m^6^A

**Figure 2.**
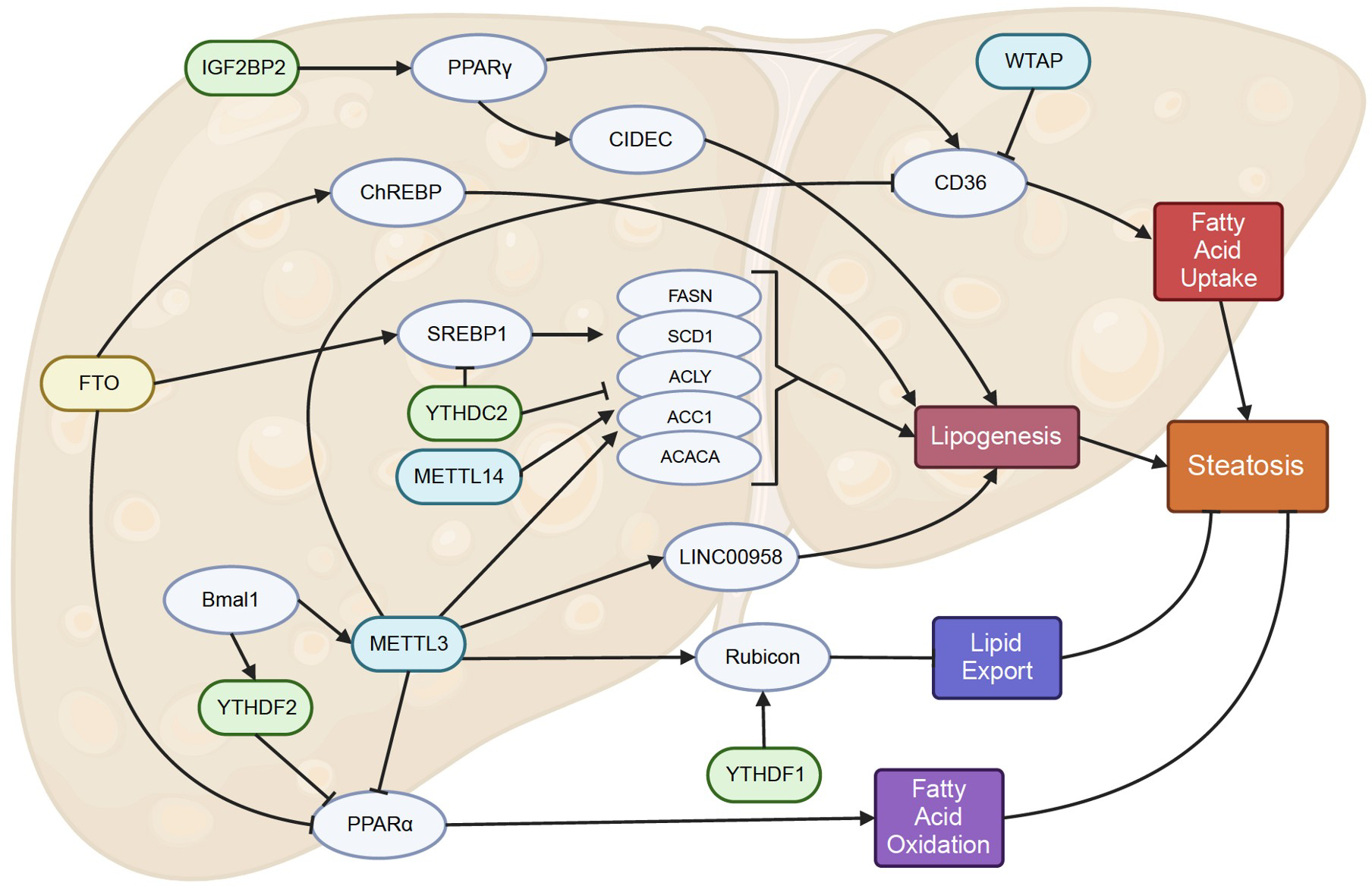
m^6^A modifications in steatosis. Writers and erasers affect the m^6^A levels on the mRNA of various genes related to fatty acid uptake, lipogenesis, lipid export, and fatty acid oxidations. Dysregulation of m^6^A-related proteins leads to dysregulation of lipid metabolism-related gene expression, which causes steatosis.

An imbalance in lipid metabolism is a significant aspect of NAFLD. Liver steatosis occurs when fatty acid uptake and *de novo* lipogenesis (DNL) exceed fatty acid oxidation and lipid export. Each of these lipid metabolism processes is affected by m^6^A modifications (Figure 2).

#### Lipogenesis and fatty acid uptake

Lipogenesis is the process in which acetyl-CoA from excess carbohydrates is converted into new fatty acids, which are then stored as triglycerides. It is largely regulated by two transcription factors: sterol regulatory binding protein 1c (SREBP-1c) and ChREBP. SREBP1c activates lipogenic genes such as fatty acid synthase (FASN), stearoyl-coenzyme A desaturase 1 (SCD1), adenosine triphosphate citrate lyase (ACLY), and acetyl-CoA carboxylase 1 (ACC1) (Ipsen et al., 2018). The expression of SREBP-1c, ChREBP, FASN, SCD1, and ACLY are all heavily affected by m^6^A modifications.

FTO, by decreasing levels of m^6^A methylation of SREBF1 and ChREBP mRNA, stabilizes their mRNA transcripts and promotes the expression of SREBF1 and ChREBP as well as downstream lipogenic genes FASN, SCD1, ACLY, and ACC1 (Tang et al., 2023). This is supported by Zhou et al. (2021), and they showed that YTHDC2, as a reader, bound to the m^6^A locations on SREBP1c, FASN, ACC1, and SCD1 mRNA and destabilized them. Glucocorticoids play a role in increasing lipid accumulation via this pathway, as the glucocorticoid receptor binds to the promoter of FTO, which leads to increased FTO levels and subsequently upregulates lipogenic genes including Srebf1, Fasn, Acaca, and Scd1 (Hu et al., 2020). Additionally, the increased FTO activity leads to the upregulation of SREBP1c, thereby promoting the activation of Cell death-inducing DFFA (DNA fragmentation factor-α)-like effector c (CIDEC), a regulator of lipid droplet size and triglyceride accumulation (Chen et al., 2018a). Conversely, a conflicting study revealed that in a NAFLD mouse model and HCC patients, SREBP1 was not upregulated. Instead, METTL3 and METTL14 directly methylated ACLY and SCD1 mRNA, resulting in enhanced expressions of ACLY and SCD1, and promoting lipogenesis (Yang et al., 2022b).

In addition to SREBP1c, m^6^A modifications also govern pathways associated with fatty acid synthesis, triglyceride synthesis, and fatty acid uptake. For instance, METTL3 stabilizes the lipogenesis-related lncRNA, LINC00958, thereby leading to the elevation of cellular cholesterol and triglyceride levels in HCC (Zuo et al., 2020). Furthermore, another study revealed that METTL3 stabilized the mRNA of diverse genes involved in glucose and lipid metabolism within the context of liver metabolic disorders and hematogenous diabetes, with Lpin1 being a notable example (Li et al., 2020). The m^6^A reader IGF2BP2, activated by Hilnc, stabilizes Pparγ mRNA, facilitating an increase in liver PPARγ level (Jiang et al., 2021b). While typically not abundantly expressed in the liver, its expression is associated with metabolic syndrome and triggers the upregulation of essential components such as fatty acid binding protein 4, Cidec, CD36, monoacylglycerol O-acyltransferase 1, and perilipin 2, which collectively contribute to the processes involved in triglyceride synthesis, free fatty acid uptake, and lipid droplet formation (Wang et al., 2020). Cd36, a key regulator of free fatty acid uptake, has also been directly governed by METTL3 and WTAP, but through an m^6^A independent mechanism. Li et al. revealed that METTL3 and WTAP inhibited Cd36 expression via histone modification (Li et al., 2021b). Additionally, the surplus of these free fatty acids might arise from increased lipoatrophy in white adipose tissues, which is regulated by WTAP suppression of IGFBP1 (Li et al., 2022c).

#### Fatty acid oxidation and lipid export

Upon uptake by hepatocytes or synthesis through DNL, fatty acids face two pathways: oxidation for energy or conversion into triglycerides. These triglycerides can then be either released into bloodstream as very low-density lipoproteins (VLDL) or stored as lipid droplets within hepatocytes.

Given that steatosis indicates the accumulation of lipid droplets, diminished fatty acid oxidation could contribute to the onset of NAFLD. Wei et al. (2022) demonstrated the involvement of FTO in the demethylation of peroxisome proliferator-activated receptor α (PPARα), a master regulator of fatty acid oxidation. The reduction in methylation of PPARα mRNA results in diminished PPARα expression, reduced fatty acid oxidation, and increased lipid accumulation. Building on this, another study confirms that PPARα undergoes methylation by METTL3 and destabilization by YTHDF2. The m^6^A methylation of PPARα constitutes an element of the Bmal1-regulated rhythmic epigenomic programming driven by the circadian clock as Bmal1 governs METTL3 and YTHDF2 levels (Zhong et al., 2018).

Beyond its impact on fatty acid oxidation, alternations in m^6^A levels also exert an influence on lipid clearance. Written by METTL3 and read by YTHDF1, the m^6^A methylation of Rubicon mRNA promotes its stability. The run domain Beclin-1-interacting and cysteine-rich domain-containing protein (RUBICON), localized to lysosomes, binds to PI2KC3 and inhibits autophagosome-lysosome fusion, thus decreasing the rate of lipid droplet clearance (Peng et al., 2022c).

### The function of m^6^A in NAFLD to NASH progression: inflammation and fibrosis

#### Inflammation

The transition from NAFLD to NASH is marked by the inclusion of inflammation and hepatocyte damage. In the context of NAFLD, the buildup of lipid droplets in hepatocytes triggers endoplasmic reticulum (ER) stress, leading to apoptosis and the subsequent release of chemokines. Notably, m^6^A methylation is intricately involved in regulating multiple aspects of these inflammatory processes.

METTL14 appears to exert a protective function against ER stress and apoptosis in hepatocytes. Wei et al. (2021) demonstrated that the unfolded protein response (UPR) promoted an augmentation in METTL14 expression. Operating an m^6^A-dependent mechanism, METTL14 facilitates the degradation of C/EBP-homologous protein (CHOP) mRNA, which in turn suppresses the expression of downstream proapoptotic target genes. This intricate process functions as a protective role against ER proteotoxic stress and the consequential liver injury. Moreover, the depletion of METTL14 results in reduced stability and translation of heat shock protein 90 beta family member 1 (Hsp90b1), endoplasmic reticulum protein 29 (Erp29), STT3 oligosaccharyltransferase complex catalytic subunit A (Stt3a), prolyl 4-hydroxylase subunit beta (P4hb), and lectin, mannose binding 1 (Lman1) mRNA transcripts. Given that these genes encode polypeptide-processing proteins, their diminished expressions can lead to an accumulation of unfolded or misfolded proteins, triggering the UPR and inducing ER stress (Cao et al., 2021).

**Figure 3.**
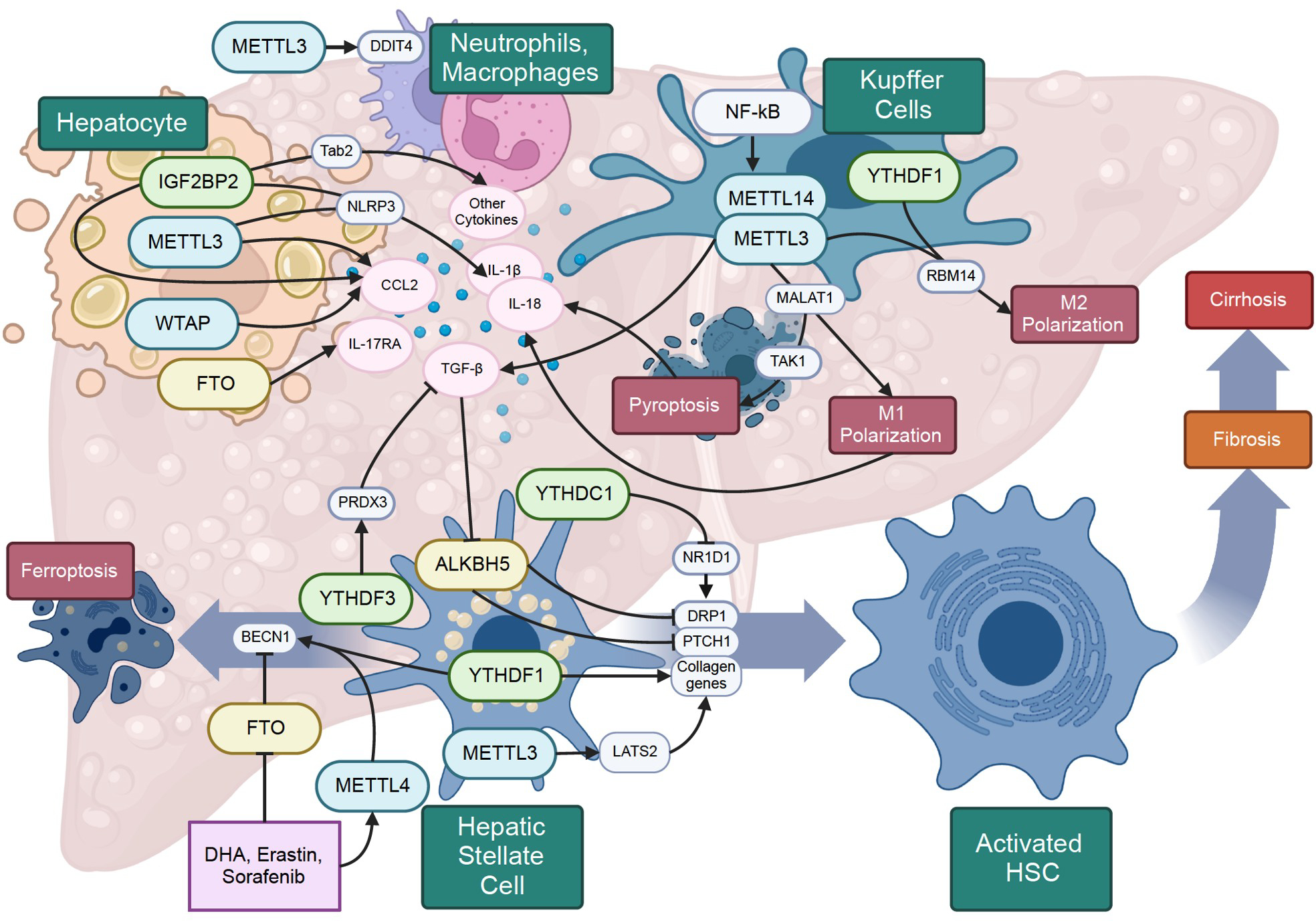
The m^6^A modifications in liver inflammation and fibrosis. Hepatocytes, hepatic stellate cells, Kupffer cells, and recruited immune cells all play a role in NASH progression. The multifaceted role of m^6^A modifications in NASH relies on its decorated mRNAs among distinct immune cell types.

Numerous proinflammatory pathways undergo regulations through m^6^A modifications. For example, TAK1-binding protein 2 (Tab2), a target of IGF2BP2, can promote the activation of JNK and NF-κB pathways, consequently leading to the expression of a range of chemokines and cytokines (Zhou et al., 2022a). Gan et al. (2022) showed that FTO, through demethylation of IL-17RA, enhanced its expression, thereby promoting liver inflammation. In the context of NASH development induced by arsenic exposure and hepatic insulin resistance, Qiu et al. (2023) found that METTL14 and IGF2BP2 contributed to the stabilization of NLRP3 mRNA, subsequently promoting NLRP3 inflammasome activation and resulting in the release of IL-1β and IL-18 (Figure 3).

The inflammatory cytokine CCL2 has been associated with the progression of steatosis to NASH (Pan et al., 2020). Within hepatocytes, METTL3 and WTAP act to inhibit CCL2 expression, whereas IGF2BP2 contributes to the elevation of CCL2 levels. Interestingly, the roles of METTL3, WTAP, and IGF2BP2 in this context may not rely solely on m^6^A modifications; instead, they might involve DNA and histone modifications as a mechanism for regulating CCL2 (Li et al., 2021b; Simon et al., 2014)

#### Immune cell activation

Stimulated by the inflammatory signals originating from distressed hepatocytes, an immune response is triggered, initiating with the liver’s resident macrophages, Kupffer cells. Feng et al. (2021) unveiled that NF-κB engaged with the promotors of METTL3 and METTL14, leading to increased m^6^A methylation across Kupffer cells. Moreover, the methylation of transforming growth factor β1 (TGF-β1) mRNA facilitates its translation.

Hepatic macrophages are classified as pro-inflammatory and cytokine-producing M1 macrophages, and the anti-inflammatory M2 macrophages. Shu et al. (2021) uncovered that METTL3 methylated and stabilized the lncRNA MALAT1, which holds significance for the polarization of M1 macrophages. Furthermore, as a consequence of the MALAT1 pathway, the engagement of TAK1 induces pyroptosis, a type of programmed cell death distinguished by the creation of cell membrane pores that release inflammatory cytokines. This aligns with another investigation where alcohol consumption-induced pyroptosis in Kupffer cells was mitigated by silencing METTL3 (Pan et al., 2023a). Additionally, METTL3 was found to potentially enhance M2 polarization in Kupffer cells through the stabilization of RBM14 mRNA facilitated by YTHDF1 (Hu et al., 2022).

The progression towards NASH is marked by the infiltration of neutrophils into the liver. Elevation in the expression of m^6^A reader IGF2BP2 within mouse hepatocytes triggers the activation of NF-κB signaling, resulting in the increased expression of Cxcl1 and Cxcl2. These changes contribute to the recruitment of neutrophils to the liver (Zhou et al., 2022a). The activation of additional myeloid cells also plays a role in the progression of NASH. Qin et al. (2021) demonstrated that METTL3 methylated and destabilized DNA damage inducible transcript 4 (DDIT4) mRNA. DDIT4 typically inhibits mTORC1 and NF-κB signaling pathways. Therefore, increased m^6^A methylation of DDIT4 caused by excessive nutrient load and metabolic ER stress results in the upregulation of mTORC1 and NF-κB signaling. This, in turn, leads to an enhancement of macrophage effector function. Taken together, the distress of hepatocytes initiates the activation of Kupffer cells via NF-κB-mediated m^6^A methylation, subsequently influencing macrophage polarization. Within the context of m^6^A methylation processes, where METTL3, METTL14 and IGFBP2 play pivotal roles, these functions encompass the safeguarding against ER stress, the regulation of proinflammatory pathways, and shaping of immune responses throughout the progression of NASH development.

#### Hepatic stellate cell activation and fibrogenesis

In the healthy liver, hepatic stellate cells (HSCs) are in a quiescent state, undergo activation by signaling from hepatocytes and macrophages, and turn into ECM-producing myofibroblasts. An investigation on the variations in m^6^A modifications across the transcriptome of mice with liver fibrosis (Fan et al., 2021) showed that among the 3,315 genes with noteworthy differences in m^6^A levels, 2,498 displayed hypermethylation, while 817 showed hypomethylation. Notably, a significant number of these genes were associated with key elements of HSC activation, including ER stress response, PPAR, and TGF-β signaling pathways. Indeed, Sun et al. (2022) revealed that peroxiredoxin 3 (PRDX3), whose translation was upregulated by m^6^A reader YTHDF3, operated as a suppressor of HSC activation and liver fibrosis through the (ROS)/TGF-β1/Smad2/3 pathway. The primary function of PRDX3 is to eliminate a significant proportion of mitochondrial hydrogen peroxide (H_2_O_2_). Therefore, a decrease in PRDX3 translation leads to an accumulation of reactive oxygen species in mitochondria, consequently triggering the activation on the TGF-β signaling pathway. It is found (Yang et al., 2022a) that TGF-β stimulation diminishes the levels of ALKBH5 in HSCs. Through its role in demethylating patched 1 (Ptch1) mRNA, ALKBH5 increases PTCH1 levels, thus impeding hedgehog signaling and subsequently reducing HSC activation. Additionally, ALKBH5 functions by eliminating the m^6^A modification on Dynamin-related protein 1 (Drp1), which in turn prevents YTHDF1-facilitated Drp1 translation. DRP1 is instrumental in driving mitochondrial fission, a pivotal process contributing to TGF-β induced HSC proliferation (Wang et al., 2023a). The m^6^A modification could indirectly influence DRP1 levels, as increased methylation of nuclear receptor subfamily 1 group d member 1 (NR1D1), a clock gene, leads to its degradation mediated by YTHDC1, thereby inhibiting the phosphorylation of DRP1 (Chen et al., 2023). Within HSCs, the reduction in DRP1 levels, arising from m^6^A methylation of either Nr1d1 or Drp1, leads to a reduced mitochondrial fission. This consequently triggers an increase of mtDNA release, activating the cGAS pathway and promoting liver fibrosis. A promising avenue emerges with the potential role of dihydroartemisinin (DHA) in alleviating liver fibrosis. DHA achieves this by orchestrating the proteasomal degradation of YTHDC1, thus contributing to the moderation of NR1D1 degradation (Chen et al., 2023) (Figure 3).

Upon activation, m^6^A modifications are essential for the perpetuation of HSC activation. The transcripts of major collagen genes undergo hypermethylation, with YTHDF1 contributing to their stability (Feng et al., 2023). Simultaneously, METTL3 engages in the modification of large tumor suppressor kinase 2 (Lats2) mRNA, an integral participant in the Hippo/Yap signaling pathway, thereby enhancing its stability. This increased LATS2 expression prompts increased phosphorylation of the downstream transcription factor YAP, resulting in the attenuation of pro-fibrotic gene expression (Li et al., 2022d). Hence, the excessive production of ECM in HSCs is due to the interplay of distinct methylation patterns, encompassing both hyper- and hypo-methylation events across various transcripts.

A prospective approach to treat liver fibrosis involves the elimination of HSCs through ferroptosis, a non-apoptotic form of programmed cell death. In this regard, m^6^A modifications play crucial roles in DHA, erastin, and sorafenib-induced ferroptosis. These compounds, known to induce ferroptosis, elicit an elevation in METTL14 levels alongside a concurrent reduction in FTO levels, and this orchestrated modulation results in an overall increase in the levels of m^6^A modifications throughout the transcriptome (Shen et al., 2021). Of notable significance is the role played by YTHDF1 in the stabilization of m^6^A decorated beclin 1 (BECN1) transcripts, a pivotal event that sets the stage for the amplification of BECN1 expression. This increased expression in turn fuels the autophagic degradation of ferritin, ultimately culminating in the execution of ferroptosis (Shen et al., 2022).

### The involvement of m^6^A function in HCC tumorigenesis

NAFLD and NASH stand as significant risk factors for the development of HCC, particularly in cases involving NASH-associated cirrhosis. Notably, in contrast to other etiological factors contributing to HCC, a substantial portion—approximately 30%—of NAFLD-related HCC cases emerge without the presence of cirrhosis, complicating the early-stage detection (Huang et al., 2021). m^6^A modifications take center stage in HCC, playing an indispensable role in tumorigenesis (Table 1). Utilizing The Cancer Genome Atlas (TCGA) database, Liu et al. (2020a) conducted an in-depth investigation that unveiled the upregulation of eleven m^6^A writers, readers, and erasers in the context of HCC. The five components within this spectrum—writers METTL3, KIAA1429, and ZC3H13, as well as readers YTHDF1 and YTHDF2—displayed a stronger correlation with worse outcomes due to their marked overexpressions.

STAT3 signaling holds a central role in obesity-driven HCC (Grohmann et al., 2018). An intriguing positive feedback loop exists between METTL3 and STAT3 in HCC, i.e., METTL3 promotes STAT3 translation through m^6^A methylation, while STAT3 facilitates the nuclear localization of METTL3 by upregulating WTAP (Liu et al., 2023a). Upon exposure to lipopolysaccharide stimulation, m^6^A modifications and the expression of GNAS undergo a substantial increase (Ding et al., 2020). The increase in GNAS expression prevents the interaction between long non-coding RNA TPTEP1 and STAT3, ultimately resulting in STAT3 downregulation. This sequence of events leads to an intensified m^6^A modification on GNAS, thereby amplifying STAT3 Y705 phosphorylation and consequently promoting the proliferation and invasion of inflammation-related HCC.

The upregulation of METTL3 levels contributes to HCC progression through multiple additional pathways. Vasculogenic mimicry (VM), a process in which aggressive tumor cells stimulate vasculogenic networks, plays a crucial role in the malignancy of HCC. METTL3 methylates and increases the translation of YAP1, thereby facilitating the development of VM by activating the hippo pathway (Qiao et al., 2021). Moreover, METTL3 is implicated in several dimensions of cellular regulation. It methylates the anti-apoptotic protein Survivin (Zhang et al., 2022b), promotes glycolysis through activating mTOR signaling (Lin et al., 2020), and contributes to the induction of epithelial-to-mesenchymal transition (EMT) by modulating Snail (Xu et al., 2020a). Additionally, METTL3 plays a pivotal role in reprograming intracellular metabolism by methylating HIF-1α mRNA, thereby promoting glycolysis and glutaminolysis (Yang et al., 2021). This in turn triggers the increase of m^6^A readers including YTHDF1. As a result, YTHDF1 facilitates the translation of autophagy-related genes like ATG2A and ATG14 to help HCC cells survive under hypoxia (Li et al., 2021a).

The increased levels of METTL3 and YTHDF1 are likely contributors to the increased translation of epidermal growth factor receptor (EGFR). This statement gains support from the study conducted by Wang et al. (2023c), wherein they observed that the upregulation of METTL3 expression resulted in Lenvatinib resistance due to the methylation of EGFR. Su et al. (2021) found that YTHDF1 promoted HCC cell viability and metastasis by facilitating the translation of EGFR. YTHDF3 also plays a role in promoting EGFR translation (Hu et al., 2023). However, an alternative study yielded contrasting results, indicating that m^6^A methylation and subsequent posttranscriptional destabilization of EGFR by METTL14 inhibit HCC cell migration, invasion, and EMT process (Shi et al., 2020). This discrepancy could be due to these m^6^A modifications being recognized by a different reader, namely YTHDF2, as elucidated by Zhong et al. (2019), who identified its role in promoting the degradation of EGFR mRNA in HCC cells. Clearly, the regulation of EGFR by m^6^A is a multifaced process influenced by numerous m^6^A readers and writers.

While HIF-1α leads to an elevation of YTHDF1 levels, HIF-2α results in a reduction of YTHDF2 levels. YTHDF2 processes the decay of interleukin 11 (IL-11) and serpin family E member 2 (SERPINE2) mRNAs. A decrease in YTHDF2 levels facilitates inflammation-mediated malignancy and disrupts vascular normalization (Hou et al., 2019). YTHDF2 is responsible for the decay of suppressor of cytokine signaling 2 (Socs2) mRNA. Consequently, the elevated METTL3 levels in HCC lead to a decrease in SOCS2 expression and an increase in tumorgenicity (Chen et al., 2018b). The last of the YTH domain family proteins, YTHDF3, is also upregulated in HCC and forms a positive feedback loop with phosphofructokinase PFKL, thereby promoting glycolysis (Zhou et al., 2022b).

Numerous other m^6^A-regulated proteins also contribute to the dynamics of HCC. For example, IGF2BP2 stabilizes flap endonuclease-1 (FEN1) transcripts methylated by METTL3, promoting liver cancer growth (Pu et al., 2020). IGF2BP1 stabilizes and ALKBH5 demethylates LY6/PLAUR domain containing 1 (LYPD1), which increases the proliferation and invasiveness of HCC cells. Notably, the decreased expression of ALKBH5 in HCC leads to an increase in LYPD1 methylation and expression (Chen et al., 2020). However, an alternative study presents a different perspective, indicating an upregulation of ALKBH5 in HCC. This study found that ALKBH5 upregulated MAP3K8 by preventing YTHDF2-mediated decay of MAP3K8 mRNA, thus promoting HCC cell proliferation, metastasis, and PD-L1^+^ macrophage recruitment (You et al., 2022). METTL14 is downregulated in HCC, and m^6^A methylation of USP48 by METTL14 plays a protective role in HCC as USP48 stabilizes SIRT6, which impedes metabolic reprogramming and glycolysis (Du et al., 2021). Upregulated WTAP destabilizes ETS proto-oncogene 1 (ETS1) transcripts by preventing them from binding to HuR, an RNA stabilizer that targets non-m^6^A modified transcripts, thus driving HCC tumor growth and proliferation (Chen et al., 2019). Similarly, upregulated KIAA1429 downregulates the tumor-suppressing gene GATA3 by adding m^6^A modifications that prevent GATA3 pre-mRNA from binding to HuR (Zhang et al., 2022c).

Further dysregulation of m^6^A modifications in HCC could potentially result from the SUMOylation of m^6^A readers and erasers. For example, the SUMOylation of Mettl3 by small ubiquitin-like modifier SUMO1 is increased upon mitogen stimulation (Xu et al., 2020a), and the induction of FTO SUMOylation at a specific site, which is caused by the activation of RANBP2 by SIRT1, promotes FTO degradation (Liu et al., 2020b).

#### Cancer stem cells

Cancer stem cells (CSCs) represent a small subset of cells within a tumor with increased tumorigenic potential compared with their more differentiated counterparts. Consequently, an increased CSC population in HCC corresponds to an enhanced cancer progression and augmented resistance to treatments. Notably, the m^6^A reader YTHDF2 is associated with poor survival rates in HCC patients. YTHDF2 recognizes m^6^A modifications on OCT4 mRNA, leading to an increase in OCT translation (Zhang et al., 2020a). As a result, this mechanism contributes to the CSC phenotype. YTHDF2 plays a distinctive role in stabilizing Frizzled-10 (FZD10) mRNA following the addition of m^6^A modifications by METTL3. This unique function holds immense importance, as increased FZD10 expression promotes the WNT/β-catenin and Hippo pathways. Consequently, this initiation leads to the transcription of specific genes that drive self-renewal, tumorigenesis, and metastasis of liver CSCs. Downstream of WNT/β-catenin is c-Jun, which in turn promotes the expression of METTL3, creating a positive feedback loop interconnecting METTL3, FZD10, β-catenin, and c-Jun. Furthermore, c-Jun activates the MAPK pathway, leading to lenvatinib resistance (Wang et al., 2023b). m^6^A modifications in CSCs are also involved in sorafenib resistance. HNF3γ, methylated by METTL14 and stabilized by IGF2BP2, promotes differentiation in both HCC cells and liver CSCs. This results in a reduction of the proportion of CSCs within HCC cells and the suppression of HCC growth. Moreover, HNF3γ promotes the expressions of OATP1B1 and OATP1B3, two major membrane transporters responsible for sorafenib uptake, thus sensitizing HCC cells to the treatment with sorafenib (Zhou et al., 2020).

FTO is also associated with HCC stemness as it promotes the expression of SRY-box transcription factor 2 (SOX2), Kruppel like factor 4 (KLF4), and NANOG by m^6^A demethylation (Bian et al., 2021). Within the scope of m^6^A modifications, RALYL, a component of the hnRNP family of m^6^A readers, contributes to the stabilization of TGF-β2 mRNA. This stabilization event subsequently activates the PI3K/AKT and STAT3 pathways and promotes HCC stemness (Wang et al., 2021a).

#### Non-coding RNAs

The scope of m^6^A modifications extends beyond mRNA, encompassing a diverse range of molecules such as lncRNAs, circRNAs, miRNAs, and rRNAs. These types of RNAs collectively contribute to the intricate landscape of HCC progression (Table 2).

Long non-coding RNAs (lncRNAs) both regulate m^6^A modifications and are affected by m^6^A modifications in HCC. For example, the lncRNA CASC11 recruits ALKBH5 to ubiquitin conjugating enzyme E2 T (UBE2T) mRNA and inhibits the interaction between UBE2T and YTHDF2, thus preventing m^6^A-dependent decay of UBE2T mRNA and promoting HCC growth and metastasis (Chen et al., 2021a). Similarly, ILF3 AS1 recruits METTL3 to ILF3 mRNA and enhances its interaction with IGF2BP2, thus promoting ILF3 mRNA stability and HCC tumorigenesis (Bo et al., 2021). AC115619 is a lncRNA that encodes the micropeptide, AC11619-22a, which binds to WTAP, preventing the assembly of the m^6^A methyltransferase complex and reducing global m^6^A levels. AC115619 is downregulated in HCC, and thus m^6^A levels are generally increased (Zhang et al., 2023b).

Increased m^6^A modifications dysregulate various lncRNAs in HCC. METTL3 and IGF2BP2 stabilize lnc-CTHCC, which binds to hnRNP K and activates YAP1 transcription, thus promoting HCC growth and metastasis (Xia et al., 2022). METTL14 and IGF2BP2 stabilize ARHGAP5-AS1, which weakens the interactions between cold shock domain containing E1 (CSDE1) and tripartite motif containing 28 (TRIM28), preventing CSDE1 degradation and coordinating oncogenic RNA regulons that activate the ERK pathway (Liu et al., 2022a). METTL16 also promotes HCC progression through destabilizing lncRNA RAB11B-AS1 (Dai et al., 2022). YTHDF2 upregulates LncAY in HCC, and through promoting BMI1 expression, lncAY activates Wnt/β-catenin signaling (Chen et al., 2021b). Increased m^6^A modifications on the lncRNA FAM111A-DT further HCC progression by promoting FAM111A transcription through interactions with YTHDC1 (Pu et al., 2023). ALKBH5 is generally downregulated in HCC, and decreased demethylation of LINC01468 increased its expression, driving NAFLD-HCC progression through CUL4A-linked degradation of SHIP2 and subsequent activation of the PI3K/AKT/mTOR signaling pathway (Wang et al., 2022b). Likewise, LINC02551, downregulated by ALKBH5, promotes HCC growth and metastasis by blocking the interaction between DEAD-box helicase 24 (DDX24) and an E3 ligase TRIM27, thus inhibiting DDX24 degradation (Zhang et al., 2022a). Overall, it appears that m^6^A modifications on lncRNAs are increased in HCC, with most m^6^A readers and writers being upregulated and erasers downregulated. However, a study by Kong et al. (2022) found that LINC01273, miR-600, and METTL3 form a feedback loop that reduces METTL3 levels in sorafenib-resistant HCC. LINC01273 stabilizes miR-600, thus increasing the repressive effect of miR-600 on METTL3 mRNA and downregulation of METTL3. METTL3 and YTHDF2 promote the degradation of LINC01273, and higher levels of LINC01273 decrease METTL3 levels thus further upregulating LINCO1273.

Several other lncRNAs also interact with miRNAs in ceRNA networks in which lncRNAs, by sponging miRNAs, prevent them from interacting with their mRNA targets. For example, LINC00958, stabilized by METTL3 methylation in HCC, upregulates hepatoma-derived growth factor (HDGF) expression by sponging miR-3619-5p, and increased HDGF promotes the expression of lipogenic genes and furthers HCC progression (Zuo et al., 2020). Similarly, METTL3 upregulates double homeobox A pseudogene 8 (DUXAP8), which, by sponging miR-584-5p, promotes MAPK1 expression and activates the MAPK/ERK pathway, causing chemoresistance in HCC (Liu et al., 2021b). METTL3 also regulates the NIFK-AS1/miR-637/AKT1 axis in the same way. Downstream of AKT1 includes MMP-7 and MMP-9, as well as OATP1B1 and OATP1B3, therefore METTL3-regulated NIFK-AS1 promotes HCC progression and Sorenifib resistance (Chen et al., 2021c). m^6^A modifications on LINC00106 by METTL3 and the subsequent stabilization by IGF2BP1 upregulate LINC00106 levels. This lncRNA sponges the miRNA let7f to activate periostin, thus promoting the properties of stemness and metastasis in HCC cells (Liang et al., 2021). METTL3-mediated m^6^A-modified lncRNAs can also downregulate them, as in the case of MEG3. Decreased sponging of miR544b by MEG3 enhances the binding of miR544b to BTG2, lowering BTG2 expression and furthering HCC progression (Wu et al., 2021). Peng et al. (2022b) found that lipopolysaccharide promoted m^6^A methylation by METTL14 of lncRNA MIR155HG, which is stabilized through a HuR-dependant pathway. By sponging miR-223, MIR155HG upregulates STAT1 and PD-L1, facilitating the immune escape of HCC cells.

m^6^A-regulated circRNAs are also able to act as ceRNAs and sponge miRNAs in HCC. CircMDK stabilizes IGF2BP1, by sponging miR-346 and miR-874-3p, it promotes the expression of ATG16L1. This activates the PI3K/AKT/mTOR signaling pathway, causing a greater proliferation of HCC cells, and an inhibition of apoptosis (Du et al., 2022). In a similar fashion, circRNA-SORE is upregulated by m^6^A modifications and activates the Wnt/β-catenin pathway by sponging miR-103a-2-5p and miR-660-3p, thus inducing Sorenifib resistance (Xu et al., 2020b). METTL3 and YTHDC1 upregulate circHPS5, which by sponging miR-270, increases HMGA2 expression, promoting EMT and CSCs in HCC (Rong et al., 2021). FTO-mediated demethylation of circGPR137B promotes its expression, and by sponging miR-4739, circGPR137B upregulates FTO. This circGPR137B/miR-4739/FTO feedback loop inhibits HCC tumorigenesis and metastasis, and circGPR137B is downregulated in HCC tissues (Liu et al., 2022b). Other circRNAs regulate m^6^A-related proteins as well. Has_circ_0008583 sponges miR-1301-3p to promote METTL3 expression (Wang et al., 2022a), and circMAP2K4 sponges miR-139-5p to promote YTHDF1 expression (Chi et al., 2021), both of which further HCC progression.

CircRNAs bind to not only miRNA but also RNA-binding proteins to regulate mRNA. ALKBH5 and YTHDF2 upregulate circCPSF6, which sustains the stability of YAP1 and drives HCC malignancy by competitively binding to PCBP2 (Chen et al., 2022). m^6^A writer KIAA1429 downregulates circDLC1, decreasing its binding to HuR and thus promoting the binding of HuR to MMP1 in HCC (Liu et al., 2021a). Another mechanism in which m^6^A modifications interact with circRNAs is by promoting the circRNA’s translation into a short peptide. The m^6^A modifications on the coding circRNA, circMAP3K4, are recognized by IGF2BP1, which promotes its translation into circMAP3K4-455aa. This peptide interacts with AIF and protects HCC cells against cisplatin-induced apoptosis, promoting cancer progression (Duan et al., 2022).

Other than lncRNAs and circRNAs, various other non-coding RNAs can be dysregulated by m^6^A modifications in HCC. For example, METTL14 interacts with the microprocessor protein DGCR8 microprocessor complex subunit (DGCR8) to promote the processing of primary microRNA 126 in an m^6^A-dependent manner, suppressing HCC progression (Ma et al., 2017). METTL5 promotes HCC tumorigenesis both *in vitro* and in mouse models. Depletion of METTL5-mediated 18S rRNA m^6^A modification results in an impaired 80S ribosome assembly and decreases the translation of fatty acid metabolism-related mRNAs (Peng et al., 2022a).

### Other RNA modifications in NAFLD-NASH-HCC progression

Although m^6^A is one of the most well-studied RNA modifications, it remains crucial to emphasize its concurrent existence alongside other modifications. In addition to m^6^A, genes related to m^5^C and m^1^A could also predict the prognosis of HCC (Li et al., 2022a). The overexpression of m^1^A writers TRMT10C, TRMT6, and ribosomal RNA processing protein 8 (RRP8) as well as m^5^A reader Y box binding protein 1 (YBX1) were correlated with poor prognosis. TRMT6 and TRMT61A-mediated m^1^A modifications on tRNA have been discovered to enhance PPARδ translation. This translation initiation subsequently stimulates cholesterol synthesis, triggering hedgehog signaling and thereby promoting the self-renewal of liver CSCs, ultimately liver tumorigenesis (Wang et al., 2021b). Additionally, Aly/REF export factor (ALYREF) has been identified as reader protein to have an oncogenic function in HCC development through directly binding to target genes by m^5^C modifications (Xue et al., 2023), while m^5^C writer, NSUN2, can enhance the stability of H19 lncRNA. This in turn leads to the recruitment of the G3BP1 oncoprotein, therefore promoting tumor development in HCC (Sun et al., 2020). The emerging functions of m^5^C, m^1^A, m^3^C, ψ and m^7^G have also surfaced in the complex landscape of HCC. This highlights the potential efficacy of diverse targeting of these modification types in cancer cells as a promising strategy for HCC treatment (Feng et al., 2022; Li et al., 2022a). The ongoing exploration of these RNA modifications, including the unfolding biological roles of m^1^A, holds substantial promise for refining our understanding of HCC progression. A crucial objective is to gain comprehensive insights into the pathophysiological functions and altered pathways governed by m^6^A, m^5^C, m^1^A, m^3^C, ψ and m^7^G in HCC, thereby unraveling their implications for therapeutic targeting.

#### Avenues for therapeutic intervention

The absence of currently effective drugs for NAFLD and NASH has prompted exploration into innovative therapeutic avenues. Approaches center around targeting RNA modifications and related processes to intervene in disease progression. Investigating m^6^A RNA modifications and their roles in inflammation, fibrosis, and metabolism offers potential intervention points. Additionally, strategies to modulate macrophage polarization through m^6^A modifications could mitigate inflammatory responses. These novel directions aim to address the urgent need for effective treatments for NAFLD and NASH.

#### Targeting RNA methylation with small molecules

Current therapeutic pursuits have predominantly concentrated on FTO and METTL3, the two most promising therapeutic enzymes. The development of inhibitors or activators for RNA methylation regulators holds significant potential in the realm of liver cancer therapy. Notable examples include STM2457, a specific METTL3 inhibitor, and the newly introduced second-generation METTL3 inhibitor, STM3006. They both show an increased potency compared with their predecessors. The rapid metabolism of STM3006 has facilitated the clinical advancement of STC15, a clinical compound maintaining similar potency and specificity (Fiorentino et al., 2023; Guirguis et al., 2023; Pan et al., 2023b; Xu et al., 2022; Yankova et al., 2021). Currently, STC15 is undergoing Phase 1 trials, which makes a significant stride in the exploration of advanced METTL3 inhibition strategies and their potential applications in cancer therapeutics (Guirguis et al., 2023).

Moreover, the potential therapeutic benefits of RNA methylation modulation through the targeting of FTO by small-molecule inhibitors are promising. Noteworthy inhibitors in this pursuit including rhein, MO-I-500, meclofenamic acid, R-2HG, fluorescein, FB23-2, and entacapone all exhibitefficacies in suppressing FTO demethylation activities (Chen et al., 2012; He et al., 2015; Huang et al., 2019; Huang et al., 2015; Peng et al., 2019; Toh et al., 2015; Wang et al., 2015). Additionally, CS1 and CS2 have been identified as portent FTO inhibitor to sensitize leukemia cells to T cell cytotoxicity (Su et al., 2020). Recent progression also highlights the specific FTO inhibitor 18097 (Xie et al., 2022), as well as small molecules targeting key players like IGF2BP2, ALKBH5, along with the ALKBH5-specific inhibitor 20m (Fang et al., 2022). Intriguingly, the impact of STM2457 on AML development and the suppressive effects of FTO inhibitor FB23-2 on AML cell proliferation highlight the complex interplay between methylation and demethylation processes (Huang et al., 2019; Yankova et al., 2021). Nevertheless, STM2457 synergizes with anti-programmed cell death protein 1 (PD-1) to reinvigorate cytotoxic CD8^+^ T cells and mediate tumor regression in NAFLD-related HCC model (Pan et al., 2023b). Targeting METTL3 using the specific inhibitor STM2457 improves the sensitivity to lenvatinib *in vitro* and *in vivo*, indicating that METTL3 may be a potential therapeutic target to overcome lenvatinib resistance in HCC (Wang et al., 2023c).

Extensive research has been dedicated to investigating numerous small molecules derived from natural products as potential regulators of RNA methylations (Bedi et al., 2020; He et al., 2015; Huang et al., 2019; Peng et al., 2019; Qiao et al., 2016; Wang et al., 2018). This exploration delves into the utilization of natural product-derived small-molecule inhibitors. Among these, Rhein stands out due to its ability to competitively bind to the active site of FTO, leading to the effective inhibition of intracellular m^6^A demethylation effectively (Chen et al., 2012). This highlights a shifting trend in modifying drugs epigenetically, which traditionally is confined to tumor-centric domains, this field is now extending its scope to leverage the broader potential of RNA biology for advancing therapeutic strategies. This evolution holds significant promise for potential applications in HCC therapy.

The growing prominence of immunotherapy underscores the pivotal role of RNA methylation within the HCC tumor microenvironment (Xu et al., 2021a; Yin et al., 2022). Notable research underscores its potential to amplify the impact of immunotherapies by influencing immune surveillance and countering immunosuppressive cell infiltration (Cai et al., 2021) (Xu et al., 2021b). Encouragingly, the targeting of m^6^A and m^5^C emerges as a potential strategy to overcome chemo- and radio-resistance in HCC, synergizing with established therapeutic approaches (Li et al., 2022b; Zhang et al., 2023a). In light of these insights, the integration of RNA methylation modulations into liver cancer therapy holds immense promise for advancing the field and ultimately enhancing patient outcomes in HCC.

#### Targeting RNA methylation in non-coding RNAs and mRNA translation

Indirect regulatory effects on translation by m^6^A-related enzymes also warrant attention. An illustrative example is METTL3’s direct activation of the PI3K-Akt-mTOR signaling pathway in retinoblastoma cancer cells (Zhang et al., 2020b). This emphasizes the complex interplay between m^6^A methylation and downstream pathways. Notably, ribosome migration, crucial for translation, can be influenced by RNA’s secondary structure and binding proteins, as exemplified by m^6^A binding protein YTHDC2 (Hsu et al., 2017; Mao et al., 2019). This intricate process potentially underlies m^6^A-facilitated translation. However, the necessity of m^6^A for YTHDC2’s roadblock-clearing function requires further elucidation (Zhang et al., 2023c). Furthermore, while current studies showcase translational regulations by m^6^A-binding proteins that impact target genes, with minimal influence on mRNA levels but significant effects on protein levels, the indirect mechanisms involved demand deeper scrutiny.

In the realm of cellular regulation, non-coding RNAs like transfer RNAs (tRNAs) and ribosomal RNAs (rRNAs) play a pivotal role in post-transcriptional control of mRNAs (Li and Mason, 2014). These non-coding RNAs are extensively modified, with the functions of these modifications still not fully elucidated. By influencing the dynamics of non-coding RNAs, these modifications could potentially impact mRNA translation, either directly or indirectly (Li and Mason, 2014). This opens intriguing possibilities for RNA-based therapeutic interventions, particularly in liver diseases like NAFLD, NASH, and HCC (Yang et al., 2023b; Zhao et al., 2020). The unique modifications found in tRNAs and rRNAs could be harnessed to modulate the translation of key mRNAs central to the progression of liver diseases (Huang et al., 2023; Zhu et al., 2023). Additionally, some m^6^A regulators oversee various RNA modifications, each with distinct regulatory functions in mRNA translation (Sun et al., 2023). The interplay between these modifications could create a composite effect, shaping the specificity of targeted mRNAs and influencing immune cell responses within the context of liver diseases (Sun et al., 2023). As we delve deeper into the intricacies of RNA modifications, there is an emerging potential to develop RNA-based therapies tailored to liver diseases. By manipulating non-coding RNAs, scientists might be able to directly influence the translation of disease-relevant mRNAs, offering a precise approach to addressing the molecular basis of liver disorders. This innovative strategy could revolutionize our treatment approaches, leading to improved outcomes for patients with NAFLD, NASH, and HCC.

### Concluding remarks

In this review, we illuminated the multifaced roles of RNA modifications, notably m^6^A, within the intricate landscape of NAFLD, NASH, and HCC. The dynamics of m^6^A within the liver exert substantial influence over critical processes including lipid metabolism, inflammation, fibrosis, and tumorigenesis. However, it is important to recognize that the impact of m^6^A is complex and context-dependent, capable of both protective and detrimental effects.

Extensive research has yielded valuable insights into the role of m^6^A in driving the progression of HCC and its associated regulatory pathways. Elevated m^6^A methylation has been identified as a driving force behind HCC advancement, exerting its influence over a multitude of key mRNAs and signaling pathways. However, the landscape in NAFLD and NASH appears less defined due to limited investigations and conflicting outcomes. Unraveling these intricate dynamics demands dedicated exploration into the function of m^6^A within HSCs, Kupffer cells and infiltrating immune cells, as well as the impact of m^6^A on non-coding RNAs specific to NAFLD and NASH. This encompasses cell-autonomous RNA modifications that influence other types of liver cell populations. It involves interactions between immune cells and hepatocytes, as well as the interplay between immune cells and HSCs, alongside the interaction between hepatocytes and HSCs. Additionally, it is crucial to investigate RNA modifications beyond m^6^A, given the limited exploration of Ψ, m^1^A, m^5^C, m^6^Am, and other modifications in the NAFLD-to-HCC progression. State-of-the-art techniques such as single-cell m^6^A sequencing offer the potential to unravel the contradictory findings concerning the influence of m^6^A. These approaches hold the possibility to shed light on the contradictory information surrounding the role of m^6^A and determine whether certain mechanisms are truly m^6^A dependent.

The potential for therapeutic interventions in NAFLD and NASH not only encompasses m^6^A-related small molecules but also extends to other potential targets including non-coding RNAs and diverse RNA modifications. This comprehensive viewpoint not only enhances our understanding but also has the potential to significantly reshape treatment approaches, offering a more promising outlook for individuals affected by these medical conditions.

Consequently, it is essential to thoroughly investigate various m^6^A targeting molecules for their potential efficacies in addressing these liver disorders. However, given the essential roles that m^6^A modifications play in regular cellular functions, a broad range of m^6^A targeting molecules might inadvertently induce undesirable side effects. Hence, a comprehensive understanding of m^6^A regulation is indispensable before designating it as a therapeutic target. Moreover, recognizing the role of m^6^A in liver diseases could also provide a valuable diagnostic tool, in addition to advancements in drug development.

## Figures and Tables

**Table 1.** mRNA m^6^A modifications in HCC

Type	Regulator	Effect of m^6^A on mRNA	Target gene	Effect of m^6^A on HCC	Ref.

Writer	METTL3	Translation	Stat3	HCC proliferation	(Liu et al., 2023a)
	METTL3	Translation	Yap1	Vasculogenic mimicry	(Qiao et al., 2021)
	METTL3	Translation	Survivin	Inhibits apoptosis	(Zhang et al., 2022b)
	METTL3	Translation	EGFR	Lenvatinib resistance, HCC cell viability and metastasis	(Wang et al., 2023c)
	METTL3	Stability	Snail	EMT	(Xu et al., 2020a)
	METTL3	Stability	FEN1	HCC growth	(Pu et al., 2020)
	METTL3	Stability	FZD10	CSCs, lenvatinib resistance	(Wang et al., 2023b)
	METTL3	Upregulation	HIF-1α	Glycolysis and glutaminolysis	(Yang et al., 2021)
	METTL14	Stability	USP48	Impedes metabolic reprogramming and glycolysis	(Du et al., 2021)
	METTL14	Stability	HNF3γ	CSC differentiation, sorafenib sensitivity	(Zhou et al., 2020)

Eraser	FTO	Decay	SOX2, KLF4, NANOG	HCC stemness	(Bian et al., 2021)
	ALKBH5	Decay	MAP3K8	HCC cell proliferation, metastasis, and macrophage recruitment	(You et al., 2022)
	ALKBH5	Stability	LYPD1	Proliferation and invasiveness of HCC cells	(Chen et al., 2020)
	WTAP	Destabilization	ETS1	HCC tumor growth and proliferation	(Chen et al., 2019)

Reader	YTHDF1	Translation	ATG2A and ATG14	HCC survival under hypoxia	(Li et al., 2021a)
	YTHDF1 YTHDF3	Translation	EGFR	Lenvatinib resistance, HCC cell viability and metastasis	(Hu et al., 2023; Su et al., 2021; Wang et al., 2023c)
	YTHDF2	Decay	IL-11	Inflammation-mediated malignancy	(Hou et al., 2019)
	YTHDF2	Decay	SERPINE2	Disruption of vascular normalization	(Chen et al., 2018b)
	YTHDF2	Decay	SOCS	Inhibits HCC cell migration, invasion, and EMT	(Chen et al., 2018b)
	YTHDF2	Decay	MAP3K8	HCC cell proliferation, metastasis, and macrophage recruitment	(You et al., 2022)
	YTHDF2	Translation	OCT4	CSC phenotype	(Zhang et al., 2020a)
	YTHDF1 YTHDF3	Translation	EGFR	Lenvatinib resistance, HCC cell viability and metastasis	(Hu et al., 2023; Su et al., 2021; Wang et al., 2023c)
	IGF2BP2	Stability	FEN1	HCC growth	(Pu et al., 2020)
	IGF2BP2	Stability	LYPD1	Proliferation and invasiveness of HCC cells	(Chen et al., 2020)
	YTHDF2	Stability	FZD10	CSCs, lenvatinib resistance	(Wang et al., 2023b)
	IGF2BP2	Stability	HNF3γ	CSC differentiation, sorafenib sensitivity	(Zhou et al., 2020)
	YTHDF1 YTHDF3	Translation	EGFR	Lenvatinib resistance, HCC cell viability and metastasis	(Hu et al., 2023; Su et al., 2021; Wang et al., 2023c)
	RALYL	Stability	TGF-β2	HCC stemness	(Wang et al., 2021a)
	HuR	Destabilization	ETS1 GATA3	HCC tumor growth, proliferation, and metastasis	(Chen et al., 2019) (Zhang et al., 2022c)

**Table 2.** Non-coding RNA networks regulated by m^6^A modifications in HCC

Type	Regulator	lncRNA/clrcRNA	miRNA or RNA binding protein	mRNA; downstream effectors	Effect in HCC	Ref.

Writer	METTL3	LINC00958	miR-3619-5p	HDGF; lipogenic genes	HCC progression	(Zuo et al., 2020)
	METTL3	DUXAP8	miR-584-5p	MAPK1; MAPK/ERK pathway	Chemoresistance	(Liu et al., 2021b)
	METTL3	NIFK-AS1	miR-637	AKT1; MMP-7/9 and OATP1B1/3	HCC progression, Sorenifib resistance	(Chen et al., 2021c)
	METTL3	MEG3	miR544b	BTG2	HCC suppression	(Wu et al., 2021)
	METTL3	LINC00106	let7f	periostin	HCC stemness and metastasis	(Liang et al., 2021)
	METTL3	circHPS5	miR-270	HMGA2	EMT, CSCs	(Rong et al., 2021)
	METTL3/14	circRNA-SORE	miR-103a-2-5p miR-660-3p	Wnt/β-catenin pathway	Sorenifib resistance	(Xu et al., 2020b)
	METTL14	MIR155HG	miR-223	STAT1 and PD-L1	Immune escape	(Peng et al., 2022b)
	METTL14	DGCR8	microRNA 126		HCC suppression	(Ma et al., 2017)
	METTL5	18S rRNA	80S ribosome assembly	fatty acid metabolism-related mRNAs	HCC oncogenic transformation	(Peng et al., 2022a)
	KIAA1429	circDLC1	HuR	MMP1		(Liu et al., 2021a)

Eraser	FTO	circGPR137B	miR-4739	FTO	Inhibition of HCC tumorigenesis and metastasis	(Liu et al., 2022b)
	FTO	circRNA-SORE	miR-103a-2-5p miR-660-3p	Wnt/β-catenin pathway	Sorenifib resistance	(Xu et al., 2020b)
	ALKBH5	circCPSF6	PCBP2	YAP1	HCC malignancy	(Chen et al., 2022)

Reader	IGF2BP1	circMDK	miR-346 miR-874-3p	ATG16L1 PI3K/AKT/mTOR pathway	HCC proliferation, apoptosis inhibition	(Du et al., 2022)
	IGF2BP1	LINC00106	let7f	periostin	HCC stemness and metastasis	(Liang et al., 2021)
	YTHDC1	circHPS5	miR-270	HMGA2	EMT, CSCs	(Rong et al., 2021)
	YTHDF1/2	circRNA-SORE	miR-103a-2-5p miR-660-3p	Wnt/β-catenin pathway	Sorenifib resistance	(Xu et al., 2020b)
	YTHDF2	circCPSF6	PCBP2	YAP1	HCC malignancy	(Chen et al., 2022)
	IGF2BP1	circMAP3K4	N/A	AIF	protects HCC cells against cisplatin-induced apoptosis	(Duan et al., 2022)

	N/A	Has_circ_0008583	miR-1301-3p	METTL3	HCC progression	(Wang et al., 2022a)
		circMAP2K4	miR-139-5p	YTHDF1	HCC progression	(Chi et al., 2021)
